# Effectiveness of alternative shock strategies for out-of-hospital cardiac arrest: A systematic review

**DOI:** 10.1016/j.resplu.2022.100232

**Published:** 2022-05-11

**Authors:** Helen Pocock, Charles D Deakin, Ranjit Lall, Christopher M Smith, Gavin D Perkins

**Affiliations:** a Warwick Clinical Trials Unit, Warwick Medical School, University of Warwick, Gibbet Hill Road, Coventry CV4 7AL, United Kingdom; b South Central Ambulance Service NHS Foundation Trust, Southern House, Sparrowgrove, Otterbourne, Winchester SO21 2RU, United Kingdom; c University Hospital Southampton NHS Foundation Trust, Tremona Road, Southampton, SO16 6YD, United Kingdom; d University Hospitals Birmingham NHS Foundation Trust, Birmingham Heartlands Hospital, Bordesley Green East, Birmingham B9 5SS, United Kingdom

**Keywords:** Defibrillation, Out-of-Hospital Cardiac Arrest, Ventricular Fibrillation, Electric Countershock, Cardiopulmonary Resuscitation

## Abstract

**Aim:**

To determine the optimal first-shock energy level for biphasic defibrillation and whether fixed or escalating protocols for subsequent shocks are most effective.

**Methods:**

We searched Medline, Embase, Cochrane CENTRAL, CINAHL, the Web of Science and national and international trial registry databases for papers published from database inception to January 2022. We reviewed reference lists of key papers to identify additional references. The population included adults sustaining non traumatic out-of-hospital cardiac arrest subject to attempted defibrillation. Studies of internal or monophasic defibrillation and studies other than randomised controlled trials or prospective cohorts were excluded. Two reviewers assessed study relevance. Data extraction and risk of bias assessment, using the ROBINS-I tool, were conducted by one reviewer and checked by a second reviewer. Data underwent intention-to-treat analysis.

**Results:**

We identified no studies evaluating first shock energy. Only one study (*n* = 738) comparing fixed versus escalating energy met eligibility criteria: a prospective cohort analysis of a randomised controlled trial of manual versus mechanical CPR. High fixed (360 J) energy was compared with an escalating (200–200/300–360 J) strategy. Researchers found 27.5% (70/255) of patients in the escalating energy group and 27.61% (132/478) in the fixed high energy group survived to hospital discharge (unadjusted risk ratio 0.99, 95% CI 0.73, 1.23). Results were of very low certainty as the study was at serious risk of bias.

**Conclusion:**

This systematic review did not identify an optimal first-shock energy for biphasic defibrillation. We identified no survival advantage at 30 days when comparing 360 J fixed with 200 J escalating strategy.

## Introduction

### Description of the condition

Out-of-hospital cardiac arrest presents a health challenge across the world.^1^ In Europe, between 11–37% of out-of-hospital cardiac arrests present with a shockable rhythm on initial assessment.^2^ The more quickly a shock can be delivered, the greater the chance of survival.^3^ Amongst witnessed cases, the chance of survival to 30 days decreases with each shock (OR 0.9: 95% CI 0.88–0.92) and survival benefit is most marked for the first three shocks.^4^

The vast majority of cardiac arrests occur outside of hospital. Evidence suggests that in-hospital cardiac arrest (IHCA) and out-of-hospital cardiac arrest (OHCA) should be considered separately.^5^ Those sustaining out-of-hospital events tend to have fewer co-morbidities, have an unwitnessed arrest and longer delays before cardiopulmonary resuscitation (CPR) is started.

### Description of the intervention and evidence of uncertainty

The intervention, attempted cardiac defibrillation, is the administration of an electric shock to a person experiencing cardiac arrest due to ventricular fibrillation or pulseless ventricular tachycardia. Key variables in attempted defibrillation are the waveform, shock energy and delivery protocol.

#### Waveforms

Devices delivering a monophasic waveform are no longer manufactured; biphasic waveforms have been shown to result in greater first shock success with fewer myocardial complications.^6^ The biphasic truncated exponential (BTE) waveform delivers a peak current which decays exponentially before reversing direction. The rectilinear biphasic (RLB) waveform maintains current at a fixed level of current in a saw-tooth waveform before reversing direction. A survey of UK Ambulance Services carried out in preparation for this work, revealed that these two waveforms are the most commonly used. An evidence review informing current international resuscitation guidelines found no evidence for the superiority of either waveform.^7^

#### First shock energy

A previous systematic review reported no difference in first shock success for selected energy levels between 120 and 200 J.^8^ However, this was based on evidence published prior to 2011, employing waveforms that are no longer in clinical use and algorithms delivering initial shocks in stacks of three.

#### Delivery protocol

Current guidelines advise delivery of a shock as early as possible in the resuscitation attempt and delivery of single, rather than stacked shocks.^9^ It is not known whether the probability of successful defibrillation remains constant after each successive shock,^10^ and hence whether, after an unsuccessful shock, subsequent shocks should be delivered at a higher or the same energy level.^6^ Current guidelines state that:

*“A range of defibrillation energy levels have been recommended by manufacturers and previous guidelines, ranging from 120-360 J. In the absence of any clear evidence for the optimal initial and subsequent energy levels, any energy level within this range is acceptable for the initial shock, followed by a fixed or escalating strategy up to maximum output of the defibrillator.”* p.6, Soar et al.^11^

### How the intervention might work

At a cellular level, VF is a re-entrant arrhythmia. When a normal wavefront reaches a conduction block, such as ischaemic tissue, the wavefront re-enters the cell and starts to propagate vortex wavefronts. These vortices create ‘daughter’ wavelets which drift around the area of tissue interacting with other wavelets creating electrical chaos.^12^ The mechanism by which this pattern of fibrillation can be halted (defibrillation) is not fully understood. A number of theories have been postulated including critical mass theory, upper limit of vulnerability and refractory period extension.^13^ During the vulnerable refractory period, an electrical stimulus may trigger the fibrillation mechanism if the strength of stimulation falls within lower and upper limits. In order to defibrillate, a shock must halt the wavefronts whilst not itself initiating further wavefronts, thus it must be above the upper limit of vulnerability.^12^ Whilst delivery of too little energy is unlikely to defibrillate the heart, too much may cause myocardial injury, manifested by asystole or ventricular arrhythmias (e.g. refibrillation).^14^

### Why it is important to do this review

It is important to understand which shock strategy will produce the best outcomes. The International Liaison Committee on Resuscitation highlight a lack of good quality evidence for optimal first-shock energy level and no strong evidence favouring either fixing subsequent shocks at the same level or escalating the energy.^6^ Much of the evidence is based on old resuscitation regimes and uses a variety of different endpoints making meaningful comparison difficult.^8^

The aim of this systematic review was to investigate evidence for the effectiveness of defibrillation strategies on adults sustaining OHCA. The specific objectives were to determine the effect of the commonly employed (1) first shock defibrillation energies, and (2) defibrillation strategies, on Return Of Organised Rhythm (ROOR), survival and neurological function at discharge/30 days.

## Methods

### Protocol and registration

We registered details of the protocol for this systematic review on the PROSPERO database at https://www.crd.york.ac.uk/PROSPERO/display_record.asp?ID=CRD42020167709. We report results according to the Preferred Reporting Items for Systematic Reviews and Meta-Analyses (PRISMA) guidance, a checklist for which can be found in Appendix A1.^15^

### Eligibility criteria and outcomes

We anticipated that the above objectives could be met by assessment of the same studies; the specific inclusion criteria are:

1. Standard vs. high first shock energy:
  • *Population:* Adults receiving external biphasic shock treatment for out-of-hospital cardiac arrest.
  • *Intervention:* Delivery of standard energy (120 J for RLB waveform and 200 J for BTE waveform).
  • *Comparison*: Delivery of high energy (150 J or 200 J for RLB waveform and 300 J or 360 J for BTE waveform).
  • *Outcomes:* Primary outcome: Return of Organised Rhythm after 1 shock
    Secondary outcomes: i. Survival to discharge/30 days.
      ii. Neurological function (modified Rankin Score) at discharge/30 days.
  • *Study type:* Randomised Controlled Trials (RCTs), quasi-RCTs, prospective observational cohort studies.
2. Fixed vs. escalating energy strategy
  • *Population:* Adults receiving external biphasic shock treatment for out-of-hospital cardiac arrest
  • *Intervention:* Standard escalating energy strategy (120–150–200 J) for RLB waveform and 200–300–360 J for BTE waveform).
  • *Comparison:* High energy fixed strategy (200–200–200 J for RLB waveform and 360–360–360 J for BTE waveform).
  • *Outcomes:* Primary outcome: Return of Organised Rhythm within 3 shocks
    Secondary outcomes: i. Survival to discharge/30 days.
      ii. Neurological function (modified Rankin Score) at discharge/30 days.
  • *Study type:* Randomised Controlled Trials (RCTs), quasi-RCTs, prospective observational cohort studies.

#### Population

Adult recommended energy levels are one-size-fits-all whereas they are weight-dependent for paediatric patients.^16^ According to guidelines, patients are treated as adults if they appear to be adults.^16^ However, research studies tend to include only over-18 s as adult; this review used the individual studies’ definition of adult.

A previous systematic review of first shock energy included only trials where the initial rhythm was shockable.^8^ We also considered studies including initially non-shockable rhythm which later converted to shockable since, according to guidelines, both groups are treated with the same shock energy.

#### Intervention

##### First shock

The European Resuscitation Council (ERC) guidelines recommend a first shock energy of at least 150 J in biphasic waveforms.^11^ This proves problematic for the RLB waveform, where the manufacturer’s pre-set first shock is 120 J.^17^ Common UK Ambulance Service practice is to start with 120 J for the RLB waveform and here it was considered as standard care. For the BTE waveform, common UK practice and manufacturer’s pre-set first shock level is 200 J.^18^.

##### Fixed versus escalating strategy

Although guidelines advise *consideration* of escalating the shock energy following a failed shock or for recurrent fibrillation,^9^ manufacturers’ recommended strategies and UK customary Ambulance Service practice is 120 J, followed by 150 J, with further shocks at 200 J for RLB waveform and 200 J, followed by 300 J with further shocks at 360 J for BTE waveform.

#### Comparison

##### First shock

We made within-waveform comparisons with the two alternative first shock energies found in UK practice. The comparator groups were 150 J/200 J for RLB waveform and 300 J/360 J for BTE waveform.

##### Fixed versus escalating strategy

We selected a fixed high-energy strategy as the comparator group. This is in current use for BTE waveforms (360 J) in many UK Ambulance Services. We also included the corresponding RLB high-energy level (200 J) for comparison.

#### Outcomes

The primary outcome was Return Of Organised Rhythm (ROOR), defined as the detection of two QRS complexes <5 s apart, <60 s after defibrillation.^19^ To compare fixed and escalating strategies, our primary outcome was ROOR after a third, but prior to a fourth, shock. Three shocks may appear an arbitrary number in the course of a resuscitation attempt, but it is the number specified in manufacturers' recommended escalation strategies.17., 18. It is also the number of shocks within which survival benefit is most marked.^4^.

**Fig. 1:**
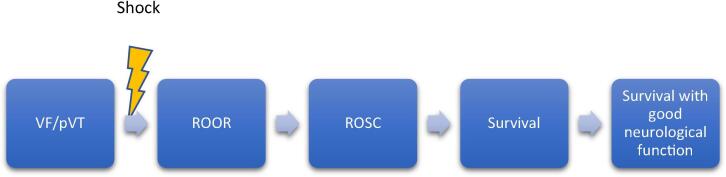
Clinical pathway following successful defibrillation.

Fig. 1 shows the necessary steps in the clinical pathway from cardiac arrest to survival following successful defibrillation.

Traditionally, shock success has been defined as termination of fibrillation (ToF) five seconds after shock delivery.^19^ However, the resultant rhythm may be asystole, a highly undesirable patient outcome. Return of spontaneous circulation (ROSC), whilst a preferred outcome, is liable to detection bias since presence of a carotid pulse is difficult to confirm.^20^

Return of organised rhythm (ROOR), is a more sensitive marker of shock success^19^ and less susceptible to detection bias, as it may be assessed retrospectively by independent reviewers.

The secondary outcomes were those prioritised in the Core Outcome Set for Cardiac Arrest (COSCA): survival and survival with a favourable neurological outcome at discharge or 30 days.^21^ These outcomes were considered separately since discharge occurs at variable timepoints.

We also report key data elements, forming the clinical and process variables from the Utstein template. Data were only presumed missing for patients reported to have survived at that time point.

#### Study type

Although studies fulfilling a randomised controlled trial (RCT) design are the gold standard for inclusion in systematic reviews, scoping work revealed few relevant RCTs or quasi-RCTs (studies where methods of allocation are not strictly random). We decided, a priori, to synthesise evidence from quasi-randomised trials separately.^22^

We included prospective observational studies, i.e. cohort studies, but case-controlled studies were excluded due to their retrospective design and hence susceptibility to further bias.^23^

### Search methods for identification of studies

The following databases were searched:

• Ovid MEDLINE ® 1946 to 15 Jan 2022
• Ovid Embase Classic + Embase 1947 to 15 Jan 2022
• CINAHL 1981 to Jan 2022
• Cochrane CENTRAL 1996 to Jan 2022
• Web of Science database 1997 to 15 Jan 2022

We performed the Cochrane-recommended strategy of searching Medline, Embase and Cochrane CENTRAL.^24^ In addition we searched CINAHL, which specifically includes literature related to Emergency Medical Systems (EMS), and Web of Science.,

We searched the following international trials databases:

• Clinical Trials.gov (https://clinicaltrials.gov/) searched on 16 Jan 2022
• WHO International Clinical Trials Registry Platform (https://apps.who.int/trialsearch/) searched on 16 Jan 2022
• ISRCTN register (https://www.isrctn.com/) searched on 16 Jan 2022

We hand searched reference lists of key papers for additional references and conducted a citation search.

Appendix A2 shows the search strategies for each electronic database**.** We included the Cochrane highly sensitive search strategy (sensitivity-maximising version, 2008 revision) to identify randomised controlled trials in Medline in the Medline search and the standard Cochrane search strategy to identify trials in Embase in the Embase search.^25^

### Study selection and data extraction

We managed references using Endnote (version X9, Clarivate Analytics, UK).^26^ De-duplication was carried out in Endnote and references then transferred to Rayyan (Qatar Computing Research Institute, Qatar) for screening.27., 28. Reviewers were not blinded to author, journal, study site or results during screening. Two reviewers (HP, CS) independently conducted primary screening of titles and abstracts against the Inclusion-Exclusion criteria checklist (see Appendix A3). Disagreement was resolved by discussion. Secondary screening of full text articles was conducted by two independent reviewers (HP, CD) and differences resolved by discussion. The reason for exclusion that was the highest in the hierarchy of exclusions listed was reported.^29^ The level of agreement between reviewers was assessed using Cohen’s kappa statistic for inter-observer variance as described by Viera and Garrett (2005).^30^

A Study Characteristics form (shown in Appendix A4) was used to collate study information.

#### Data synthesis

We took an intention-to-treat approach when considering shock strategy. This is because not all patients require or receive more than one shock. This objective was designed to pragmatically identify the best shock strategy to adopt rather than to compare the efficacy of subsequent shocks.

We extracted binary outcomes and report risk ratios with a 95% confidence interval. Risk ratios are the preferred relative effect measure for cohort studies as they are more intuitive, being a direct measure of outcome probability.^31^ Data were collected by one reviewer, using the Data Extraction Form (shown in Appendix A5), and 10% checked by a second reviewer.

## Results

### Study selection and characteristics

**Fig. 2:**
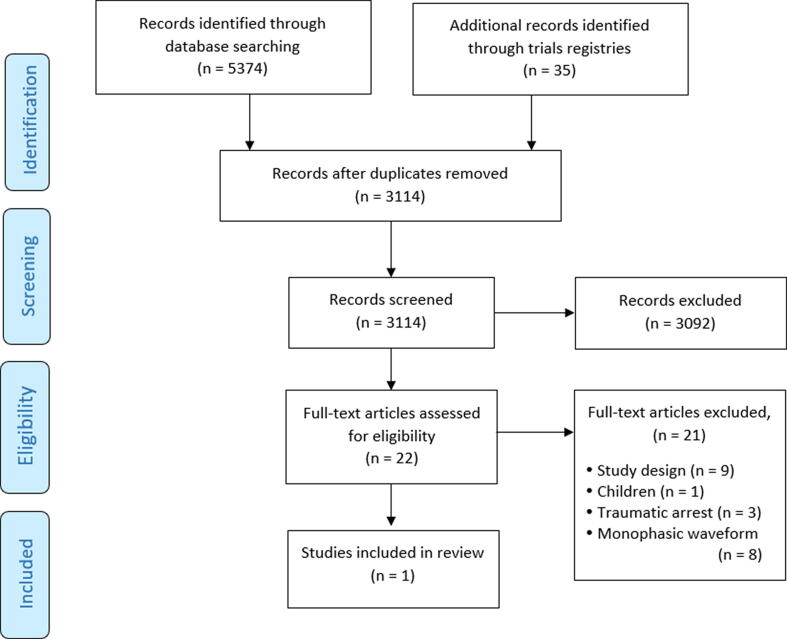
PRISMA diagram of included studies.

We conducted the search in November 2019 and updated it in January 2022. These are summarised in the PRISMA study flow diagram, see Fig. 2.^32^

We identified no additional studies through hand searching, or citation searching. In all, 3114 unique references were identified. We identified no papers requiring translation into English. On primary review, 3092 (99%) studies were excluded (kappa = 0.5 indicating moderate agreement, see Appendix A6). The updated search identified 300 unique references, all of which were excluded by title and abstract screening. Full text review of 22 studies excluded 21 studies (kappa = 1.0) resulting in the identification of only one study that met eligibility criteria. Numbers of articles excluded for each reason at second-level screen are recorded on the PRISMA flow diagram and details can be found in Appendix A7.

### First shock energy

We identified no studies which compared a standard shock with a high energy first shock.

### Fixed versus escalating shock strategy

We identified a single prospective cohort study. The study was a secondary analysis of a randomised controlled trial comparing manual versus mechanical CPR.^33^ Findings are summarised in Appendix A8.

Olsen et al.^33^ found no significant difference in survival to hospital discharge (unadjusted risk ratio 0.99, 95% CI 0.73, 1.23) in the escalating energy group (27.5%, 70/255) compared to the fixed high-energy group (27.6%, 132/478). Study authors took account of clustering by site by including the site as a random effect in the analysis. There were differences in the baseline characteristics of participants between the intervention and control group. Patients in the escalating group were slightly older than those in the fixed energy group (64 years versus 62 years respectively). Bystander CPR was more frequently performed in the escalating than the fixed group (68% versus 46% respectively) and patients waited significantly longer for an EMS response (8 min versus 6 min respectively). These differences did not yield any significant difference in the results when adjusted for these potential confounders.

The recruiting sites varied in their use of the high fixed (360 J) strategy (one site) versus escalating (200–200/300–360 J) strategy (four sites) which formed the intervention and comparator groups. Within the escalating energy group, some defibrillators delivered a 200 J second shock whilst others delivered 300 J and the proportion of each is not specified. There was also some variability in CPR protocol with 3-minute CPR cycles being delivered in the Netherlands and 2-minute cycles elsewhere; it is not clear what proportion of the analysed cohort received each.

### Risk of bias

Risk of bias was independently assessed by two reviewers using the Risk Of Bias In Non-randomised Studies of Interventions (ROBINS-I) tool, shown in Appendix A9.^34^ We judged the study to be at serious risk of bias due to potential for confounding of the effect of the intervention as randomisation was clustered by site. Only one site used the fixed high energy strategy and so there could have been something about the population or intervention delivery that acted as hidden confounders. The study was also at serious risk of bias for missing data. For 17.5% of patients, we could not determine whether fixed or escalating energy was received so it is possible that a disproportionate number of patients’ data may have been missing from one arm. For those whose energy level was known, survival data were missing for 5 patients which was 0.67% of the overall sample but 1.92% of the escalating energy arm. Study authors undertook a complete case analysis of survival data but reasons for missing data were not cited in the paper. A risk of bias table is presented (Table 1).

**Table 1:** Risk of bias assessment.

### Certainty of the evidence

We rated the evidence, according to GRADE methodology, as having very low certainty (see Appendix A9). Since it comprised a single observational study, certainty was initially rated low and further rated down for serious risk of bias and imprecision.35., 36., 37.

## Discussion

We present two key findings from this systematic review. For the research question comparing a standard- versus high-energy first shock strategy, no relevant evidence was identified. For the research question comparing fixed versus escalating energy levels for subsequent shocks, we identified only a single observational study, involving 738 participants who were enrolled in a randomised controlled trial comparing different chest compression strategies. This study was assessed as being very low certainty evidence (having been downgraded for serious risk of bias and imprecision). The study reported no difference in survival to hospital discharge between groups, this being the only *a priori* identified outcome sought in this review.

The first European Resuscitation Council guidelines advocated delivery of a shock sequence of 200–200–360 J.^38^ This was based on early animal and clinical investigations suggesting a defibrillation threshold for success between 175 and 400 J, below which success was unlikely and re-initiation of VF a possibility, but above which structural and functional damage to the myocardium was likely.^38^

Within the animal literature, paediatric porcine models of short-duration VF (<1 minute) broadly support guideline shock energies of 2–4 J/kg,39., 40. with the biphasic waveforms achieving D90 (90% probability of shock success) within this range (2.9 J/kg for RLB and 3.4 J/kg for BTE waveforms).^41^ When higher shock energies were explored, no difference in outcome was found between doses of 2, 3, or 7 J/kg.^42^ An *adult* swine model employing a 6-minute VF period, better reflecting out-of-hospital cardiac arrest, found no significant difference in restoration of circulation or measures of harm (changes in left ventricular end diastolic function or troponin levels) when 150 J and 360 J shock energies were compared (5/8 (62%) vs. 7/11 (63%) for 150 J and 360 J respectively).^43^ Animal studies do not suggest benefit with higher shock energies.

Within the paediatric literature, a key case series of 27 children established 2 J/kg as the recommended shock energy using monophasic waveform.^44^ Three observational studies using monophasic waveform suggested greater benefit using a higher shock energy although these studies were small and their designs heterogenous due to the studies’ settings (out-of-hospital/in-hospital/both settings respectively).45., 46., 47. Within the dose range 0.5 – 5 J/kg, a prospective observational in-hospital study using *biphasic* waveform (*n* = 48), identified 2.5–3 J/kg as the most successful dose for achieving ROSC.^48^ When including both in- *and* out-of-hospital studies, the energy range 1.7–2.5 J/kg was associated with higher rates of survival to discharge compared to higher or lower energy ranges.^49^ A randomised controlled trial in the out-of-hospital setting found that patients displaying ROOR at 60 s had received lower first shock energies than those not displaying ROOR (1.47 [0.93–2.32] J/kg vs. 4.18 [3.12–5.08] J/kg) although this did not translate to a difference in survival to hospital.^50^ No clear evidence of benefit with higher shock energies is therefore provided by the paediatric literature.

Evidence surrounding adult in-hospital cardiac arrest is scarce. A small study of both in- and out-of-hospital arrests detected no significant difference in VF termination between fixed (150–150–150 J) and escalating (100–150-200 J) biphasic waveform strategies until the third shock of 200 J, which produced significantly improved outcomes.^51^ More recently, a multicentre randomised trial of adult IHCA found no significant difference in outcome (ToF/ROSC or survival to 24hrs/7days/30 days) between 150 J fixed energy and higher energy escalating strategies (200–300–360 J) when comparing either first shock or overall strategy.^52^

Though a different condition with a different cause and prognosis, studies of cardioversion of atrial fibrillation may offer some indirect evidence. The randomised controlled CHESS trial found that a high fixed strategy (360 J) produced better first shock success and required fewer shocks overall than a low escalating strategy (125–150–200 J), with no difference in adverse effects.^53^

A systematic review conducted by Morrison et al.^8^ found no difference in first shock success for biphasic shock energies of 120–200 J.^8^ Despite the fact that the included studies incorporated stacked shock protocols with less emphasis on high-quality CPR, and compared biphasic with monophasic energies, the question of optimal defibrillation energy within the context of more recent resuscitation guidelines seems to have attracted little research attention. Studies such as TIMBER and BIPHASIC were instrumental in establishing 150 J and 200 J as acceptable BTE first shock energies and are still relevant to current guidelines.9., 54., 55. EMS providers are increasingly exploring higher first- and subsequent-shock energies without the accompanying high quality evidence to support such strategies.33., 56.

The ERC guidelines advise that neither a fixed nor an escalating strategy is supported by the evidence and so either are acceptable.^9^ This review supports that assertion. A minimum first shock energy for biphasic devices of 150 J for RLB and 200 J for BTE is recommended although using the highest setting of the device is also acceptable.^9^ We have found no evidence to dispute this recommendation.

One might speculate that since shock success for the biphasic defibrillation waveform has been reported between 81.8% and 100%,57., 58., there is little need to further differentiate between shock energies. However, the outcome measure, termination of fibrillation, is de-emphasised in the Core Outcome Set for Cardiac Arrest (COSCA) since it is not meaningful to patients and public.^21^ The more important outcomes – return of an organised rhythm/spontaneous circulation and survival - have featured less in the defibrillation literature, although survival featured in the single eligible study. That this study is very recent is encouraging and suggests that further investigation is both warranted and timely. Our review of clinical trial registries revealed no future clinical trials addressing this issue other than the feasibility study planned by this group.^59^

### Limitations

It may be that we failed to identify studies that have been conducted and reported. The research questions were very specific, based on current UK practice, and the choice of narrow, focused PICO questions was deliberate. A broader question could have been asked, for example comparing fixed versus escalating strategies without specifying the shock energies, however the resultant answer would have been of limited clinical utility.^60^

We may have retrieved relevant studies but subsequently excluded them as the eligibility criteria may have been overly restrictive. When eligibility criteria were applied to the 3114 records identified for primary screening, 77% (*n* = 2405) addressed the wrong outcome, intervention or population. Review articles and retrospective cohort studies were common amongst the excluded records. Amongst the excluded retrospective studies, none made between-biphasic-waveform comparisons. Three papers reported biphasic BTE versus monophasic waveform comparisons. One study provided no information regarding energy levels, a second combined different energy protocols within the waveform and a third did not specify how many shocks had been delivered.61., 62., 63. The latter two studies included lower energy levels than those sought by this review (150 J BTE). Three papers reported studies with no comparator group. Two studies explored transthoracic impedance: the first utilised fixed-energy 150 J BTE shocks, terminating fibrillation in 90% of cases following initial shock, and 99% after three shocks.^64^ In the second, an initial shock of 200 J BTE terminated fibrillation in 93% of cases.^10^ Taken together, these studies suggest favouring lower energy shocks, however termination of fibrillation may result in rhythms either capable or incapable of sustaining a pulse. In a study exploring ventricular fibrillation waveform features, initial shocks of 120 J RLB achieved return of organised rhythm in 27% of cases.^65^ As well as being liable to selection bias, the retrospective observational studies offer little towards answering the research questions.

### Research recommendations

We have made a specific recommendation following the findings of this review. We present this in Table 2 following the EPICOT + format.^66^ In summary, there is a need for an adequately powered randomised controlled trial to compare biphasic first shock energies and subsequent shock strategies in out-of-hospital cardiac arrest.

**Table 2:** Research recommendations.

Core elements		Issues to consider	Research recommendation
E	Evidence	What is the current evidence?	One observational study conducted in the out-of-hospital setting
P	Population	Diagnosis, disease stage, comorbidity, risk factor, sex, age, ethnic group, specific inclusion or exclusion criteria, clinical setting	Adults receiving external biphasic shock treatment for out-of-hospital cardiac arrest
I	Intervention	Type, frequency, dose, duration, prognostic factor	Delivery of biphasic shocks using escalating strategy (120–150-200 J for RLB waveform and 200–300-360 J for BTE waveform)
C	Comparison	Placebo, routine care, alternative treatment/ management	Delivery of biphasic shocks using high energy fixed strategy (200–200–200 J RLB waveform and 360–360–360 J for BTE waveform)
O	Outcomes	Which clinical or patient related outcomes will the researcher need to measure, improve, influence or accomplish? Which methods of measurement should be used?	Conversion to ROOR, Survival, Neurological function (mRS)
T	Time stamp	Date of literature search or recommendation	January 2022
d	Disease burden		
T	Timeliness	Time aspects of core elements:	
		Mean age of population	Adults
		Duration of intervention	Out-of-hospital phase of cardiac arrest management
		Length of follow-up	Up to one year
s	Study type	What is the most appropriate study design to address the proposed question?	Randomised controlled trial

## Conclusion

We did not identify any studies comparing first shock biphasic defibrillation energies in this systematic review. One study provided very low certainty evidence of no difference between fixed and escalating energy strategies.

## Conflict of interests

HP reports no conflicts of interest.

CDD reports no conflicts of interest.

RL reports no conflicts of interest.

CMS is an NIHR Clinical Lecturer in Emergency Medicine. He has volunteer roles with Resuscitation Council UK, European Resuscitation Council (ERC) and International Liaison Committee on Resuscitation (ILCOR).

GDP holds editor roles with Resuscitation and Resuscitation Plus.

## CRediT authorship contribution statement

**Helen Pocock:** Methodology, Formal analysis, Investigation, Funding acquisition. **Charles D Deakin:** Methodology, Investigation, Supervision, Funding acquisition. **Ranjit Lall:** Methodology, Formal analysis, Supervision, Funding acquisition. **Christopher M Smith:** Investigation. **Gavin D Perkins:** Methodology, Supervision, Funding acquisition.
