# Supplementary material for: Effectiveness of alternative shock strategies for out-of-hospital cardiac arrest: A systematic review
Source: Resusc Plus. 2022 May 11;10:100232. doi: 10.1016/j.resplu.2022.100232 (PMC9114679; doi:10.1016/j.resplu.2022.100232)
Supplement: Supplementary data 1 [file mmc1.docx]

## Appendix A1

### PRISMA checklist

| **Section and Topic** | **Item #** | **Checklist item** | **Location where item is reported** |
| --- | --- | --- | --- |
| **TITLE** | | |  |
| Title | 1 | Identify the report as a systematic review. | Title; Abstract; Introduction: Why it is important to do this review, paragraph (para.) 1 Methods: Protocol & registration (para. 1, sentence (sen.) 1. |
| **ABSTRACT** | | |  |
| Abstract | 2 | See the PRISMA 2020 for Abstracts checklist. | Abstract. |
| **INTRODUCTION** | | |  |
| Rationale | 3 | Describe the rationale for the review in the context of existing knowledge. | Introduction. |
| Objectives | 4 | Provide an explicit statement of the objective(s) or question(s) the review addresses. | Introduction: Why it is important to do this review: sen. 2. |
| **METHODS** | | |  |
| Eligibility criteria | 5 | Specify the inclusion and exclusion criteria for the review and how studies were grouped for the syntheses. | Methods: Eligibility criteria and outcomes:, Population, Intervention, Comparison, Outcomes, Study type; Appendix A3. |
| Information sources | 6 | Specify all databases, registers, websites, organisations, reference lists and other sources searched or consulted to identify studies. Specify the date when each source was last searched or consulted. | Methods: Search methods for identification of studies. |
| Search strategy | 7 | Present the full search strategies for all databases, registers and websites, including any filters and limits used. | Appendix A2. |
| Selection process | 8 | Specify the methods used to decide whether a study met the inclusion criteria of the review, including how many reviewers screened each record and each report retrieved, whether they worked independently, and if applicable, details of automation tools used in the process. | Methods: Study selection and data extraction; Appendix A4. |
| Data collection process | 9 | Specify the methods used to collect data from reports, including how many reviewers collected data from each report, whether they worked independently, any processes for obtaining or confirming data from study investigators, and if applicable, details of automation tools used in the process. | Methods: Outcome measures, Study selection and data extraction; Appendix A5. |
| Data items | 10a | List and define all outcomes for which data were sought. Specify whether all results that were compatible with each outcome domain in each study were sought (e.g. for all measures, time points, analyses), and if not, the methods used to decide which results to collect. | Methods: Outcomes, Appendix A5. |
|  | 10b | List and define all other variables for which data were sought (e.g. participant and intervention characteristics, funding sources). Describe any assumptions made about any missing or unclear information. | Appendix A4; Methods: Outcomes: para. 7, sen. 3. |
| Study risk of bias assessment | 11 | Specify the methods used to assess risk of bias in the included studies, including details of the tool(s) used, how many reviewers assessed each study and whether they worked independently, and if applicable, details of automation tools used in the process. | Results: Risk of bias. |
| Effect measures | 12 | Specify for each outcome the effect measure(s) (e.g. risk ratio, mean difference) used in the synthesis or presentation of results. | Methods: Data synthesis, para.2. |
| Synthesis methods | 13a | Describe the processes used to decide which studies were eligible for each synthesis (e.g. tabulating the study intervention characteristics and comparing against the planned groups for each synthesis (item #5)). | N/A |
|  | 13b | Describe any methods required to prepare the data for presentation or synthesis, such as handling of missing summary statistics, or data conversions. | N/A |
|  | 13c | Describe any methods used to tabulate or visually display results of individual studies and syntheses. | N/A |
|  | 13d | Describe any methods used to synthesize results and provide a rationale for the choice(s). If meta-analysis was performed, describe the model(s), method(s) to identify the presence and extent of statistical heterogeneity, and software package(s) used. | N/A |
|  | 13e | Describe any methods used to explore possible causes of heterogeneity among study results (e.g. subgroup analysis, meta-regression). | N/A |
|  | 13f | Describe any sensitivity analyses conducted to assess robustness of the synthesized results. | N/A |
| Reporting bias assessment | 14 | Describe any methods used to assess risk of bias due to missing results in a synthesis (arising from reporting biases). | N/A |
| Certainty assessment | 15 | Describe any methods used to assess certainty (or confidence) in the body of evidence for an outcome. | N/A |
| **RESULTS** | | |  |
| Study selection | 16a | Describe the results of the search and selection process, from the number of records identified in the search to the number of studies included in the review, ideally using a flow diagram. | Results: Study selection and characteristics; Figure 2; Appendix A7. |
|  | 16b | Cite studies that might appear to meet the inclusion criteria, but which were excluded, and explain why they were excluded. | Appendix A7; Limitations. |
| Study characteristics | 17 | Cite each included study and present its characteristics. | Results: Fixed versus escalating shock energy |
| Risk of bias in studies | 18 | Present assessments of risk of bias for each included study. | Results: Risk of bias: Table 1; Appendix A8. |
| Results of individual studies | 19 | For all outcomes, present, for each study: (a) summary statistics for each group (where appropriate) and (b) an effect estimate and its precision (e.g. confidence/credible interval), ideally using structured tables or plots. | Results: Fixed versus escalating shock energy; Appendix A9. |
| Results of syntheses | 20a | For each synthesis, briefly summarise the characteristics and risk of bias among contributing studies. | N/A |
|  | 20b | Present results of all statistical syntheses conducted. If meta-analysis was done, present for each the summary estimate and its precision (e.g. confidence/credible interval) and measures of statistical heterogeneity. If comparing groups, describe the direction of the effect. | N/A |
|  | 20c | Present results of all investigations of possible causes of heterogeneity among study results. | N/A |
|  | 20d | Present results of all sensitivity analyses conducted to assess the robustness of the synthesized results. | N/A |
| Reporting biases | 21 | Present assessments of risk of bias due to missing results (arising from reporting biases) for each synthesis assessed. | N/A |
| Certainty of evidence | 22 | Present assessments of certainty (or confidence) in the body of evidence for each outcome assessed. | Results: Quality of the evidence.  Appendix 9. |
| **DISCUSSION** | | |  |
| Discussion | 23a | Provide a general interpretation of the results in the context of other evidence. | Discussion |
|  | 23b | Discuss any limitations of the evidence included in the review. | Discussion: para.1. |
|  | 23c | Discuss any limitations of the review processes used. | Limitations. |
|  | 23d | Discuss implications of the results for practice, policy, and future research. | Discussion: Research recommendations, Table 2. |
| **OTHER INFORMATION** | | |  |
| Registration and protocol | 24a | Provide registration information for the review, including register name and registration number, or state that the review was not registered. | Methods: Protocol and registration. |
|  | 24b | Indicate where the review protocol can be accessed, or state that a protocol was not prepared. | Methods: Protocol and registration. |
|  | 24c | Describe and explain any amendments to information provided at registration or in the protocol. | N/A |
| Support | 25 | Describe sources of financial or non-financial support for the review, and the role of the funders or sponsors in the review. | Funding. |
| Competing interests | 26 | Declare any competing interests of review authors. | Conflicts of interest. |
| Availability of data, code and other materials | 27 | Report which of the following are publicly available and where they can be found: template data collection forms; data extracted from included studies; data used for all analyses; analytic code; any other materials used in the review. | Appendices. |

*From:*  Page MJ, McKenzie JE, Bossuyt PM, Boutron I, Hoffmann TC, Mulrow CD, et al. The PRISMA 2020 statement: an updated guideline for reporting systematic reviews. BMJ 2021;372:n71. doi: 10.1136/bmj.n71

For more information, visit: <http://www.prisma-statement.org/>

### PRISMA abstract checklist

| **Section and Topic** | **Item #** | **Checklist item** | **Reported (Yes/No)** |
| --- | --- | --- | --- |
| **TITLE** | | |  |
| Title | 1 | Identify the report as a systematic review. | Yes |
| **BACKGROUND** | | |  |
| Objectives | 2 | Provide an explicit statement of the main objective(s) or question(s) the review addresses. | Yes |
| **METHODS** | | |  |
| Eligibility criteria | 3 | Specify the inclusion and exclusion criteria for the review. | Yes |
| Information sources | 4 | Specify the information sources (e.g. databases, registers) used to identify studies and the date when each was last searched. | Yes |
| Risk of bias | 5 | Specify the methods used to assess risk of bias in the included studies. | Yes |
| Synthesis of results | 6 | Specify the methods used to present and synthesise results. | Yes |
| **RESULTS** | | |  |
| Included studies | 7 | Give the total number of included studies and participants and summarise relevant characteristics of studies. | Yes |
| Synthesis of results | 8 | Present results for main outcomes, preferably indicating the number of included studies and participants for each. If meta-analysis was done, report the summary estimate and confidence/credible interval. If comparing groups, indicate the direction of the effect (i.e. which group is favoured). | Yes |
| **DISCUSSION** | | |  |
| Limitations of evidence | 9 | Provide a brief summary of the limitations of the evidence included in the review (e.g. study risk of bias, inconsistency and imprecision). | Yes |
| Interpretation | 10 | Provide a general interpretation of the results and important implications. | Yes |
| **OTHER** | | |  |
| Funding | 11 | Specify the primary source of funding for the review. | Yes |
| Registration | 12 | Provide the register name and registration number. | Yes |

*From:*  Page MJ, McKenzie JE, Bossuyt PM, Boutron I, Hoffmann TC, Mulrow CD, et al. The PRISMA 2020 statement: an updated guideline for reporting systematic reviews. BMJ 2021;372:n71. doi: 10.1136/bmj.n71

For more information, visit: <http://www.prisma-statement.org/>

## Appendix A2

### Database search strategies

**Database: Ovid MEDLINE(R)**

1 out of hospital.mp.

2 out-of-hospital.mp.

3 pre hospital.mp.

4 pre-hospital.mp.

5 prehospital.mp.

6 community.mp.

7 Ambulances/

8 ambulance.mp.

9 paramedic*.mp.

10 emergency responders/ or emergency medical technicians/

11 emergency medical services.mp. or Emergency Medical Services/

12 1 or 2 or 3 or 4 or 5 or 7 or 8 or 9 or 10 or 11

13 cardiac arrest.mp.

14 heart arrest.mp.

15 heart arrest/ or death, sudden, cardiac/

16 cardio-pulmonary arrest.mp.

17 cardiopulmonary arrest.mp.

18 resuscitation.mp.

19 cardio-pulmonary resuscitation.mp.

20 cardiopulmonary resuscitation.mp.

21 Cardiopulmonary Resuscitation/

22 CPR.mp.

23 BLS.mp.

24 ALS.mp.

25 Advanced cardiac life support.mp.

26 ventricular fibrillation.mp.

27 ventricular tachycardia.mp.

28 Tachycardia, Ventricular/ or ventricular tachycardia.mp.

29 ventricular arrhythmia*.mp.

30 shockable rhythm*.mp.

31 13 or 14 or 15 or 17 or 18 or 19 or 20 or 21 or 22 or 23 or 24 or 25 or 26 or 27 or 28 or 29 or 30

32 12 and 31

33 Out-of-Hospital Cardiac Arrest/ or OHCA.mp.

34 32 or 33

35 defibrillat*.mp.

36 Defibrillators/

37 electric countershock.mp.

38 Electric Countershock/

39 electric defibrillation.mp.

40 electroversion therapy.mp.

41 cardiac electroversion.mp.

42 cardioversion.mp.

43 35 or 36 or 37 or 38 or 39 or 40 or 41 or 42

44 randomized controlled trial.pt.

45 controlled clinical trial.pt.

46 randomized.ab.

47 placebo.ab.

48 drug therapy.fs.

49 randomly.ab.

50 trial.ab.

51 groups.ab.

52 cohort studies/ and prospective studies/

53 44 or 45 or 46 or 47 or 48 or 49 or 50 or 51 or 52

54 exp animals/ not humans.sh.

55 53 not 54

56 (paediatric* or pediatric* or child*).mp.

57 55 not 56

58 34 and 42 and 57

**Database: Embase Classic+Embase**

1 out of hospital.mp.

2 out-of-hospital.mp.

3 prehospital.mp.

4 pre-hospital.mp.

5 pre hospital.mp.

6 community.mp.

7 ems.mp.

8 exp ambulance/

9 paramedic*.mp.

10 emergency responder*.mp.

11 rescue personnel/ or emergency medical technician*.mp.

12 1 or 2 or 3 or 4 or 5 or 6 or 7 or 8 or 9 or 10 or 11

13 heart arrest/ or cardiopulmonary arrest/ or sudden cardiac death/

14 cardiac arrest.mp.

15 cardiopulmonary arrest.mp.

16 cardio-pulmonary arrest.mp.

17 heart arrest.mp.

18 cardiopulmonary resuscitation.mp.

19 cardio-pulmonary resuscitation.mp.

20 resuscitation/

21 resuscitation.mp.

22 CPR.mp.

23 BLS.mp.

24 ALS.mp.

25 Advanced cardiac life support.mp.

26 exp heart ventricle fibrillation/

27 ventricular fibrillation.mp.

28 exp heart ventricle tachycardia/

29 ventricular tachycardia.mp.

30 heart ventricle arrhythmia/

31 ventricular arrhythmia*.mp.

32 shockable rhythm*.mp.

33 13 or 14 or 15 or 16 or 17 or 18 or 19 or 20 or 21 or 22 or 23 or 24 or 25 or 26 or 27 or 28 or 29

or 30 or 31 or 32

34 12 and 33

35 exp "out of hospital cardiac arrest"/ or OHCA.mp.

36 34 or 35

37 exp defibrillator/

38 exp electric countershock/

39 defibrillat*.mp.

40 defibrillation/ or defibrillator/

41 electric defibrillation.mp.

42 cardiac electroversion.mp.

43 electroversion therapy.mp.

44 cardioversion.mp.

45 37 or 38 or 39 or 40 or 41 or 42 or 43 or 44

46 crossover-procedure/ or double-blind procedure/ or randomized controlled trial/ or single-

blind procedure/ or (random* or factorial* or crossover* or cross over* or placebo* or (doubl*

adj blind*) or (singl* adj blind*) or assign* or allocat* or volunteer*).tw.

47 prospective cohort.mp.

48 prospective study/

49 47 or 48 or 49

50 (exp animal/ or nonhuman/) not exp human/

51 49 not 50

52 exp juvenile/ not exp adult/

53 51 not 52

54 36 and 45 and 53

**Database: Cochrane CENTRAL**

ID Search

#1 (out of hospital or out-of-hospital or prehospital or pre-hospital):ti,ab,kw

#2 MeSH descriptor: [Ambulances] in all MeSH products

#3 #1 or #2

#4 (cardiac arrest or heart arrest):ti,ab,kw

#5 MeSH descriptor: [Heart Arrest] explode all trees

#6 MeSH descriptor: [Death, Sudden, Cardiac] explode all trees

#7 (ventricular arrhythmia or ventricular fibrillation or ventricular tachycardia or shockable rhythm*):ti,ab,kw

#8 MeSH descriptor: [Ventricular Fibrillation] explode all trees

#9 MeSH descriptor: [Tachycardia, Ventricular] explode all trees

#10 #4 or #5 or #6 or #6 or #7 or #8 or #9

#11 MeSH descriptor: [Cardiopulmonary Resuscitation] explode all trees

#12 (cardiopulmonary resuscitation or cardio-pulmonary resuscitation or CPR or BLS or ALS):ti,ab,kw

#13 #11 or #12

#14 #10 or #13

#15 #3 and #14

#16 (defibrillat*):ti,ab,kw

#17 MeSH descriptor: [Electric Countershock] explode all trees

#18 (electric cardioversion or cardiac electroversion):ti,ab,kw

#19 #16 or # 17 or #18

#20 #15 and #19

#21 (paediatric* or pediatric* or child*):ti,ab,kw

#22 #20 not #21

**Database: CINAHL**

S1 "out of hospital" or "out-of-hospital"

S2 "prehospital" or "pre-hospital" or "pre hospital"

S3 "community"

S4 (MH "Prehospital Care") OR (MH "Rapid response (Emergency Care)")

S5 "ems"

S6 (MH "Ambulances") OR "ambulance*"

S7 (MH "Emergency Medical Technicians") OR "paramedic"

S8 S1 OR S2 OR S3 OR S4 OR S5 OR S6 OR S7

S9 (MH "Heart Arrest") OR "heart arrest"

S10 "cardiac arrest"

S11 (MH "Resuscitation") OR (MH "Resuscitation, Cardiopulmonary")

S12 "CPR" or "cardiopulmonary resuscitation" or "cardiopulmonary resuscitation"

S13 (MH "Advanced Cardiac Life Support")

S14 (MH "Ventricular Fibrillation") OR (MH "Arrhythmia, Ventricular")

S15 "ventricular fibrillation" or "VF"

S16 (MH "Tachycardia, Ventricular") OR "ventricular tachycardia"

S17 "shockable rhythm*"

S18 S9 OR S10 OR S11 OR S12 OR S13 OR S14 OR S15 OR S16 OR S17

S19 S8 AND S18

S20 “OHCA”

S21 S19 OR S20

S22 (MH "Defibrillation") OR (MH "Defibrillators, Automated External") OR (MH

"Defibrillators") OR "defibrillat*"

S23 "electroversion"

S24 (MH "Cardioversion") or "cardioversion"

S25 S22 OR S23 OR S24

S26 (MH "Randomized Controlled Trials")

S27 “randomi*ed controlled trial or RCT”

S28 (MH "Prospective Studies")

S29 (MH "Concurrent Prospective Studies")

S30 S26 OR S27 OR S28 OR S29

S31 S21 AND S25 AND S30

S32 (AG paediatric) OR (AG pediatric) OR (AG children)

S33 S31 not S32

************************

**Database: Web of Science**

#1 TS=("out of hospital" OR "out-of-hospital" OR "prehospital" OR "pre hospital" OR "pre- hospital" OR "community" OR "paramedic*" OR "emergency medical technician*" OR "ems" OR "ambulance*" OR "OHCA")

#2 TS=("cardiac arrest" OR "cardiopulmonary resuscitation" OR "heart arrest" OR "cardiopulmonary resuscitation" OR "cardio-pulmonary resuscitation" OR "cpr" OR "bls" OR "als" OR "advanced cardiac life support" OR "ventricular fibrillation" OR "ventricular tachycardia" OR “ventricular arrhythmia*” OR "shockable rhythm*")

#3 TS=("defibrillat*" OR "electric countershock" OR "cardioversion" OR "cardiac electroversion")

#4 #1 AND #2 AND #3

**Database: ClinicalTrials.gov**

Study type: All studies

Condition: Out-of-hospital cardiac arrest

Other terms: Defibrillation

**Database: WHO International Clinical Trials Registry Platform**

"out of hospital" OR "out-of-hospital" OR "prehospital" OR "pre hospital" OR "pre-hospital" OR "community" OR "paramedic*" OR "emergency medical technician*" OR "ems" OR "ambulance*" OR “OHCA”

AND "cardiac arrest" OR "cardiopulmonary resuscitation" OR "heart arrest" OR "cardio- pulmonary resuscitation" OR "cpr" OR "bls" OR "als" OR "advanced cardiac life support" OR "ventricular fibrillation" OR "ventricular tachycardia" OR "shockable rhythm"

AND "defibrillat*" OR "electric countershock" OR "cardioversion" OR "cardiac electroversion”

**Database: ISRCTN register**

Condition: Out-of-hospital cardiac arrest

Interventions: Defibrillation

*********************************************************************************************************

## Appendix A3

### Inclusion-exclusion checklist

| **Inclusion criteria** | **Exclusion criteria** |
| --- | --- |
| - Adult patients (patients treated using adult defibrillation protocol) - Out-of-hospital cardiac arrest with a non-traumatic aetiology - External biphasic defibrillation attempted - Randomised controlled trials - Quasi-randomised controlled trials (any study using method of allocation that is not strictly random e.g. varying allocation according to day of the week) - Prospective cohort studies - Studies evaluating first shock energy, or fixed versus escalating strategy, for defibrillation | - Animal models - Children (patients treated using paediatric defibrillation protocol i.e. shock energy according to weight) - In-hospital cardiac arrest - Traumatic arrest - Internal defibrillation (implantable cardiac defibrillators) - Monophasic defibrillation - Study designs other than RCT or prospective cohort - Foreign language papers for which an English translation cannot be obtained from the author |

## Appendix A4

### Characteristics of study form

| Study ID: | | Source (page no./fig etc.) |
| --- | --- | --- |
| Methods | *Study type:*  *Country of study:*  *Dates of data collection:*  *Resus guidelines followed:*  *Randomisation methods:*  *Blinding:*  *Loss to follow-up:* |  |
| Participants | *N=*  *Assigned per group:*  *Assessed at final data point (per group):*  *Sex of patients:*  *Age of patients (median/mean):*  *Inclusion criteria:*  *Exclusion criteria:*  *% witnessed arrest:*  *% receiving BCPR:*  *% initially shockable rhythm:*  *Call to EMS arrival:*  *Time to first defib:* |  |
| Interventions | *Defib type:*  *Waveform:*  *First shock energy:*  *Shock strategy:* |  |
| Outcomes  reported (full range and time points) |  |  |
| Notes |  |  |

## Appendix A5

### Data Extraction Form

| **Reference:** |  |
| --- | --- |

**Return of an organised rhythm (ROOR)**

| **Outcome** | **Timepoint** | **Shock strategy** | | | | | | |
| --- | --- | --- | --- | --- | --- | --- | --- | --- |
|  |  | **120-150-200** | | **150-200-200** | | **200-200-200** | | |
|  |  | Events | Total | Events | Total | | Events | Total |
| **ROOR** | After 1^st^ shock* |  |  |  |  | |  |  |
|  | Within 3 shocks |  |  |  |  | |  |  |

*before next shock

**Survival**

| **Outcome** | **Timepoint** | **Shock strategy** | | |
| --- | --- | --- | --- | --- |
|  |  | **120-150-200** | **150-200-200** | **200-200-200** |
|  | Discharge or 30 days |  |  |  |

**Neurological function: mRS score**

| **Outcome** | **Timepoint** | **Shock strategy** | | | |
| --- | --- | --- | --- | --- | --- |
|  |  | **mRS** | **120-150-200** | **150-200-200** | **200-200-200** |
|  | Discharge or 30 days | **0** |  |  |  |
|  |  | **1** |  |  |  |
|  |  | **2** |  |  |  |
|  |  | **3** |  |  |  |
|  |  | **4** |  |  |  |
|  |  | **5** |  |  |  |
|  |  | **6** |  |  |  |

## Appendix A6

### Calculation of kappa statistic

|  |  | Reviewer 2 | | |
| --- | --- | --- | --- | --- |
|  |  | Included | Excluded | **Total** |
| Reviewer 1 | Included | 11 | 18 | 29 |
|  | Excluded | 9 | 2776 | 2785 |
|  | **Total** | 20 | 2794 | 2814 |

Expected agreement, Pₑ = {(20/2814) x (29/2814)} + {(2794/2814) x (2785/2814)}

= (0.007 x 0.01) + (0.993 x 0.99)

= 0.98

Observed agreement, P₀ = (11 + 2776) / 2814

= 2787/2814

= 0.99

Kappa = (0.99 - 0.98) / (1 - 0.98)

= 0.01/ 0.02

= 0.5

## Appendix A7

### Reasons for exclusion

###

| **Reference** | **Reason for exclusion** | | | | | | |
| --- | --- | --- | --- | --- | --- | --- | --- |
|  | Animals | Children | IHCA | Traumatic arrest | Internal defibrillation | Monophasic defibrillation | Study design |
| Gliner & White (1999) |  |  |  |  |  |  | x |
| Hagihara et al (2018) |  |  |  |  |  | x |  |
| Hasegawa et al (2015) |  |  |  | x |  |  |  |
| Hess et al (2008) High peak current… |  |  |  |  |  |  | x |
| Hess et al (2008) Increased prevalence… |  |  |  |  |  | x |  |
| Hess et al (2011) |  |  |  |  |  |  | x  Shocks for Initial vs. recurrent VF |
| Jost et al (2010) |  |  |  |  |  |  | x  Stacked vs. single shocks |
| Kajino et al (2009) |  |  |  |  |  | x |  |
| Kaneko et al (2003) |  |  |  |  |  |  | x  Abstract only |
| Ko et al (2004) |  |  |  | x |  |  |  |
| Kudenchuk et al (2006) |  |  |  |  |  | x |  |
| Martens et al (2001) |  |  |  |  |  | x |  |
| Mochmann et al (2014) |  |  |  |  |  |  | x  Abstract only |
| Morrison et al (2005) |  |  |  |  |  | x |  |
| Poole et al (1997) |  | x |  |  |  |  |  |
| Rea et al (2005) |  |  |  |  |  |  | x |
| Schneider et al (2000) |  |  |  |  |  | x |  |
| Stiell et al (2007) |  |  |  |  |  |  | x |
| Tanabe et al (2012) |  |  |  | x |  |  |  |
| van Alem et al (2003) |  |  |  |  |  | x |  |
| Walker et al (2009) |  |  |  |  |  |  | x |

## Appendix A8

### Summary of Findings tables

#### Objective 1: First shock energy

| **200J vs 360J first shock energy for out-of-hospital defibrillation in adults** | | | | | | |
| --- | --- | --- | --- | --- | --- | --- |
| **Population:** Adults receiving external biphasic shock treatment for out-of-hospital cardiac arrest  **Intervention:** Delivery of initial biphasic BTE waveform shock at 200J  **Comparison:** Delivery of initial BTE shock at 360J | | | | | | |
| **Outcomes** | **Illustrative comparative risks (95% CI)** | | **Relative effect (95% CI)** | **No. of participants (& studies)** | **Quality of evidence (GRADE)** | **Comments** |
|  | **Assumed risk** | **Corresponding risk** |  |  |  |  |
|  | **360J (control)** | **200J (intervention 1)** |  |  |  |  |
| **Return of an organised rhythm (ROOR)** after 1^st^ shock |  |  |  |  |  |  |
| **Survival** to discharge/30 days |  | No data for this objective |  |  |  |  |
| **Good neurological function** **(mRS score 0-3)** at discharge/30 days |  |  |  |  |  |  |
| **Footnotes** | | | | | | |

#### Objective 2: Shock strategy

| **200-300-360J vs 360-360-360J strategy for out-of-hospital defibrillation in adults** | | | | | | |
| --- | --- | --- | --- | --- | --- | --- |
| **Population:** Adults receiving more than one external biphasic shock for out of hospital cardiac arrest  **Intervention:** Biphasic RLB waveform strategy of 200-300-360J  **Comparison:** Biphasic RLB waveform strategy of 360-360-360J | | | | | | |
| **Outcomes** | **Illustrative comparative risks (95% CI)** | | **Relative effect (95% CI)** | **No. of participants (& studies)** | **Quality of evidence (GRADE)** | **Comments** |
|  | **Assumed risk** | **Corresponding risk** |  |  |  |  |
|  | **360-360-360J (control)** | **200-300-360J (intervention 1)** |  |  |  |  |
| **Return of an organised rhythm (ROOR)** within 3 shocks |  |  | No data for this outcome |  |  |  |
| **Survival** to discharge/30 days | 132/478 = **27.61%** | 70/255 = **27.45%** | **0.99** (0.73 to 1.23) | 733  (1) | Very low* |  |
| **Good neurological function** **(mRS score 0-3)** at discharge/30 days |  | No data for this outcome |  |  |  |  |
| **Footnotes**  ***GRADE assessment:**  ***Risk of Bias:*** Downgraded for 2 x crucial within-study limitations;  ***Inconsistency:*** None;  ***Indirectness:*** Although there was a slight difference in resuscitation guidelines applied to the treatment arms (3-minute CPR cycles being  delivered in the Netherlands and 2-minute cycles elsewhere) the evidence has not been downgraded as this was thought to have no more  impact on the outcome than differences in post-resuscitation care which were  not accounted for.  ***Imprecision:*** Downgraded as the number of participants in the review was lower than that generated by a sample size calculation for a trial  (Assuming current survival 0.2, aim to increase this by 10%. 2-sided study with α 0.05, sample size = 6510)  ***Publication bias:*** Downgraded for low number of included studies. | | | | | | |

## Appendix A9

**ROBINS-I tool (Stage II): For each study Study ID:**

Olsen et al (2019) post-hoc analysis of CIRC data

**Specify a target randomised trial specific to the study:**

| Design | Individually randomised / Cluster randomised / Matched (e.g. cross-over) |
| --- | --- |
| Participants | Adults receiving at least one external biphasic shock for out-of-hospital cardiac arrest |
| Experimental intervention | 360J fixed strategy |
| Comparator | 200-300-360J strategy |

**Is your aim for this study…?**

**✓** to assess the effect of *assignment to* intervention

□ to assess the effect of *starting an adhering to* intervention

**Specify the outcome**

| Survival to discharge |
| --- |

**Specify the numeric result being assessed**

| AOR = 0.81 (95% CI, 0.54 – 1.22), p = 0.32 |
| --- |

**Risk of bias assessment**

| **Bias domain** | **Signalling questions** | **Comments** | **Response options** |
| --- | --- | --- | --- |
| Bias due to confounding | 1.1 Is there potential for confounding of the effect of intervention in this study?  **If N/PN to 1.1:** the study can be considered to be at low risk of bias due to confounding and no further signalling questions need be considered | Each site used just one shock strategy. Lots of sites used the escalating strategy; only one used the fixed strategy, therefore there could be something about the latter population that is different from the others, e.g. baseline health, socioeconomic status, ethnicity  p.116, col 1, para 2: …there may be hidden confounders such as socioeconomic status and comorbidities that might explain why more shocks were necessary in the higher energy group | Y / PY / PN / N |
|  | **If Y/PY to 1.1**: determine whether there is a need to assess time-varying confounding: | | |
|  | 1.2. Was the analysis based on splitting participants’ follow up time according to intervention received?  **If N/PN**, answer questions relating to baseline confounding (1.4 to 1.6)  **If Y/PY**, proceed to question 1.3. |  | NA/ Y / PY / PN / N / NI |
|  | 1.3. Were intervention discontinuations or switches likely to be related to factors that are prognostic for the outcome?  **If N/PN**, answer questions relating to baseline confounding (1.4 to 1.6)  **If Y/PY**, answer questions relating to both baseline and time-varying confounding (1.7 and 1.8) |  | NA/ Y / PY / PN / N / NI |
|  | **Questions relating to baseline confounding only:** | | |
|  | 1.4. Did the authors use an appropriate analysis method that controlled for all the important confounding domains? | p.114, col 1, para 3: Logistic regression analysis adjusted for confounding effects of age, witnessed arrest, cardiac arrest location and response interval. | NA/ Y / PY / PN / N / NI |
|  | 1.5. **If Y/PY to 1.4**: Were confounding domains that were controlled for measured validly and reliably by the variables available in this study? | Age ✓  Witnessed event, location - taken from patient records.  Response interval - objectively measured.  Sites had QA process in place for collecting this data | NA/ Y / PY / PN / N / NI |
|  | 1.6. Did the authors control for any post-intervention variables that could have been affected by the intervention? | p.117, col 2, para 1: Post intervention drugs differed between groups. Did not control for this. | NA/ Y / PY / PN / N / NI |
|  | **Questions relating to baseline and time-varying confounding:** | | |
|  | 1.7**.** Did the authors use an appropriate analysis method that adjusted for all the important confounding domains and for time-varying confounding? | Could not switch between interventions as different sites used different energy strategies (each site used one energy strategy | NA/ Y / PY / PN / N / NI |
|  | 1.8. **If Y/PY to 1.7**: Were confounding domains that were adjusted for measured validly and reliably by the variables available in this study? |  | NA/ Y / PY / PN / N / NI |
|  | **Risk of bias judgement** | | Low / Moderate / Serious / Critical / NI |
| Bias in selection of participants into the study | 2.1. Was selection of participants into the study (or into the analysis) based on participant characteristics observed after the start of intervention?  **If N/PN to 2.1:** go to 2.4  2.2. **If Y/PY to 2.1**: Were the post-intervention variables that influenced selection likely to be associated with intervention?  2.3 **If Y/PY to 2.2**: Were the post-intervention variables that influenced selection likely to be influenced by the outcome or a cause of the outcome? | Patients included in analysis in table 2, p.115 if their shock strategy was known | Y / PY / PN / N / NI  NA/ Y / PY / PN / N / NI  NA/ Y / PY / PN / N / NI |
|  | 2.4. Do start of follow-up and start of intervention coincide for most participants? |  | Y / PY / PN / N / NI |
|  | 2.5. **If Y/PY to 2.2 and 2.3, or N/PN to 2.4**: Were adjustment techniques used that are likely to correct for the presence of selection biases? |  | NA/ Y / PY / PN / N / NI |
|  | **Risk of bias judgement** | | Low / Moderate / Serious / Critical / NI |
| Bias in classification of interventions | 3.1 Were intervention groups clearly defined? | Eligibility criteria p.113, col 1, para 5. 200-300-360J v. 360-360-360J | Y / PY / PN / N / NI |
|  | 3.2 Was the information used to define intervention groups recorded at the start of the intervention? | Yes, presenting rhythm, p.113, col 1, para 5 | Y / PY / PN / N / NI |
|  | 3.3 Could classification of intervention status have been affected by knowledge of the outcome or risk of the outcome? | Shock energies delivered recorded by defibs | Y / PY / PN / N / NI |
|  | **Risk of bias judgement** |  | Low / Moderate / Serious / Critical / NI |
| Bias due to deviations from intended interventions | **If your aim for this study is to assess the effect of assignment to intervention, answer questions 4.1 and 4.2** | | |
|  | 4.1. Were there deviations from the intended intervention beyond what would be expected in usual practice? | No option for paramedics to choose a defib with different energy settings as they were all set the same at site level. May have been possible to change the energy manually (not normal practice) or for a different energy level to be delivered by a different provider (normal practice). P.113, col 2, para 3: ‘patients who did not meet the fixed or escalating strategy were excluded’ but it is not clear how many of the 160 patients excluded were due to intentional deviation | Y / PY / PN / N / NI |
|  | 4.2. **If Y/PY to 4.1**: Were these deviations from intended intervention unbalanced between groups *and* likely to have affected the outcome? | This was not a study of shock energy but a post-hoc analysis of a study of mechanical chest compression | NA/ Y / PY / PN / N / NI |
|  | **If your aim for this study is to assess the effect of starting and adhering to intervention, answer questions 4.3 to 4.6** | | |
|  | 4.3. Were important co-interventions balanced across intervention groups? |  | Y / PY / PN / N / NI |
|  | 4.4. Was the intervention implemented successfully for most participants? |  | Y / PY / PN / N / NI |
|  | 4.5. Did study participants adhere to the assigned intervention regimen? |  | Y / PY / PN / N / NI |
|  | 4.6. **If N/PN to 4.3, 4.4 or 4.5**: Was an appropriate analysis used to estimate the effect of starting and adhering to the intervention? |  | NA/ Y / PY / PN / N / NI |
|  | **Risk of bias judgement** | | Low / Moderate / Serious / Critical / NI |
| Bias due to missing data | 5.1 Were outcome data available for all, or nearly all, participants? | Survival data available for all included patients | Y / PY / PN / N / NI |
|  | 5.2 Were participants excluded due to missing data on intervention status? | p.114, col 1, para 4: 160/912 = 17.5% could not be categorised into fixed/escalating | Y / PY / PN / N / NI |
|  | 5.3 Were participants excluded due to missing data on other variables needed for the analysis? | All other information was reported | Y / PY / PN / N / NI |
|  | 5.4 **If PN/N to 5.1, or Y/PY to 5.2 or 5.3**: Are the proportion of participants and reasons for missing data similar across interventions? | A disproportionate number of participants may have been excluded from one arm but no data to confirm/refute this | NA/ Y / PY / PN / N / NI |
|  | 5.5 **If PN/N to 5.1, or Y/PY to 5.2 or 5.3**: Is there evidence that results were robust to the presence of missing data? | No missing data | NA/ Y / PY / PN / N / NI |
|  | **Risk of bias judgement** | | Low / Moderate / Serious / Critical / NI |
| Bias in measurement of outcomes | 6.1 Could the outcome measure have been influenced by knowledge of the intervention received? | Survival recorded by the hospital | Y / PY / PN / N / NI |
|  | 6.2 Were outcome assessors aware of the intervention received by study participants? | Hospital staff did not review defib records | Y / PY / PN / N / NI |
|  | 6.3 Were the methods of outcome assessment comparable across intervention groups? | Survival | Y / PY / PN / N / NI |
|  | 6.4 Were any systematic errors in measurement of the outcome related to intervention received? |  | Y / PY / PN / N / NI |
|  | **Risk of bias judgement** | | Low / Moderate / Serious / Critical / NI |
| Bias in selection of the reported result | Is the reported effect estimate likely to be selected, on the basis of the results, from...  7.1. ... multiple outcome *measurements* within the outcome domain? | Survival at various standard (in terms of OHCA research) time-points collected and reported | Y / PY / PN / N / NI |
|  | 7.2 ... multiple *analyses* of the intervention-outcome relationship? | Adjusted and unadjusted reported | Y / PY / PN / N / NI |
|  | 7.3 ... different *subgroups*? |  | Y / PY / PN / N / NI |
|  | **Risk of bias judgement** | | Low / Moderate / Serious / Critical / NI |
